# UDRNet: unsupervised deformable registration network of lung CT images with hybrid attention mechanism

**DOI:** 10.3389/fmed.2025.1689742

**Published:** 2025-10-15

**Authors:** Xida Ma, Mingyu Wang, Limin Zhang, Zhuo Liu, Yongshun Zhao

**Affiliations:** The First Affiliated Hospital of Dalian Medical University, Dalian, China

**Keywords:** medical image registration, unsupervised learning, lung cancer diagnosis, hybrid attention mechanism, encoder-decoder

## Abstract

With the continuous updates and iterations of diagnostic equipment and technologies, the diagnosis of lung diseases has shifted from single-time-point imaging to multi-time-point imaging data, and from single-modal diagnostic data to multi-modal diagnostic data. However, during this process, factors such as respiratory motion and organ deformation pose challenges for tracking the same lung lesion across multiple time points or modalities, as well as for observing its progression trends. Therefore, to address the challenge of tracking the same lesion region in lung images across different states, we proposes an unsupervised deformable registration network of lung CT images with hybrid attention mechanism. The model directly predicts the deformation vector field (DVF) through an end-to-end encoder-decoder architecture, solving the problems of time consumption and dependence on annotated data in traditional methods. Specifically, we design a Spatial and Channel Hybrid Attention Fusion Module (scHAF) to fuse shallow spatial and channel features in skip connections, enhancing the model's semantic alignment ability and improving the learning of registration-relevant region features. Meanwhile, we design an unsupervised training strategy that optimizes the model using image similarity loss, avoiding the reliance on real deformation field labels. Finally, extensive experiments on the CT Lung Registration dataset demonstrate that our model outperforms baseline methods like 3D VoxelMorph in metrics such as Dice (54.92%), NCC (91.49%), and MSE (89.90%). Further ablation experiments confirm the effectiveness of modules such as scHAF.

## 1 Introduction

Medical image registration is a key technology in medical image analysis, aimed at aligning anatomical structures between images by finding the optimal spatial transformation parameters. It is widely applied in clinical diagnosis and treatment, such as surgical guidance (1–3), disease treatment, and tracking (4, 5). Through medical image registration, clinicians are able to compare and analyze lesion locations and changes, and explore the trends and severity of patient conditions, thereby improving the accuracy of disease diagnosis and the rationality of treatment plan formulation (6). Among the various applications of medical image registration, lung CT image registration is one of the most necessary and important use cases (7, 8). This is because the lung is a complex and highly elastic organ, and its internal anatomical structures undergo complex deformations due to factors such as respiration, organ motion, as well as equipment and patient posture during imaging. These deformations can vary in magnitude and some tissues may undergo significant changes. For example, during radiation therapy for lung cancer, the tumor's motion range can reach several centimeters (9). By registering CT images at different respiratory phases, the tumor's movement trajectory can be quantified, ensuring that the radiation therapy covers all potential tumor locations while avoiding excessive radiation to surrounding healthy tissues caused by tumor displacement.

However, with the development of big data technology and advancements in imaging equipment, acquiring lung CT image data itself is no longer a significant challenge. However, large amounts of lung CT images often face issues such as uneven data quality and missing labels. Additionally, two CT images of the same lung cancer patient may have inconsistencies in imaging equipment parameters and pose deviations. This necessitates the use of medical image registration technology to align the patient's CT images, allowing clinicians to observe changes in the patient's lung lesion area across multiple CT scans and accurately assess the disease or formulate treatment plans. Existing medical image registration research still has certain limitations in addressing these issues. Therefore, to address these challenges, we propose a deformable lung CT image registration model based on unsupervised learning. By establishing an end-to-end registration architecture, the model performs lung CT image registration while designing a hybrid attention mechanism to achieve semantic fusion and alignment of shallow and deep features. Moreover, an unsupervised learning strategy is used to train the model on a large number of unlabeled lung CT images.

Our contributions in this paper are three-fold:

1. We propose a deformable lung CT image registration model (UDRNet )based on unsupervised learning. This model adopts an encoder-decoder structure and directly predicts the deformation vector field (DVF) between image pairs through an end-to-end learning approach, thereby achieving efficient unsupervised registration. The model is particularly well-suited for handling complex deformation issues in lung CT images, such as tissue changes caused by respiratory motion and image discrepancies resulting from inconsistencies in imaging equipment parameters.
2. We designed the Spatial and Channel Hybrid Attention Fusion Module (scHAF), which combines shallow spatial and channel features at skip connections to encourage better semantic fusion and alignment, allowing the model to learn the underlying relationships between the images to be registered and the deformation field. This hybrid attention mechanism not only retains the information from shallow features that are more relevant to registration but also enhances the model ability to capture features by weighting both spatial and channel dimensions.
3. We conducted extensive experiments on the CT Lung Registration dataset to validate the effectiveness of the proposed model and the scHAF module. The experimental results show that our model outperforms or is comparable to several other advanced registration methods across various registration metrics. Specifically, after incorporating the scHAF module, the model showed improvements in all evaluation metrics. These results demonstrate the significant impact of the scHAF module in enhancing registration accuracy and robustness, especially when dealing with complex lung CT images, as it can more accurately capture the changes in lung structures, thereby improving both registration accuracy and robustness.

## 2 Related works

### 2.1 Conventional medical image registration methods

Traditional registration methods are based on local similarity between images, manually computing the deformation field from the image to be registered to the registered image. This approach is highly time-consuming and labor-intensive (10). Researchers are exploring faster, more robust, and more general methods. Deep learning approaches use deep neural networks with strong inclusivity and fitting capabilities (11, 12). Deep learning-based lung image registration methods can leverage trained models to directly predict the deformation vector field between image pairs through a single forward pass, thus completing the registration in a short time. Additionally, deep learning-based methods can overcome the issue of lacking corresponding features in multimodal registration tasks, as they can learn task-specific features without requiring strict prior definitions. While many deep learning-based methods have shown registration accuracy comparable to traditional methods in certain tasks, there are still several challenges in clinical applications. These include the high cost and errors in data labeling, computational efficiency in model training and inference, improving model generalization ability, the interpretability of model decision-making processes, the complexity of multimodal image registration, the lack of training datasets, and the issue of derivative smoothing in the optimization process. These problems limit the effectiveness and reliability of deep learning-based registration technologies in clinical applications, requiring further research to address them.

One approach is to borrow from the research paradigm of optical flow methods, treating the non-rigid registration problem as a diffusion process by continuously estimating the driving deformation to achieve the alignment force vector (13), known as the Demons algorithm. This method has undergone numerous deformable and improved variations (14–16) and is used in the well-known open-source project ITK (The Insight Segmentation and Registration Toolkit) (17). A method sharing the differential diffeomorphic mapping hypothesis with the Demons algorithm is Large Deformation Diffeomorphic Metric Mapping (LDDMM) (18), which focuses on differential metric mapping under large deformations and formally proves the existence of a minimization function under smoothness assumptions, making an important step forward in large deformation registration. However, for organs such as the lungs, which experience significant and frequent motion, methods under the large deformation assumption, although effective in fitting anatomical structures affected by abrupt changes such as respiratory motion, still cannot handle deformations like sliding motion (19). To address this, some methods use segmentation of the lung mask or chest structures during registration. This allows for separately smoothing the tangential and normal components near the lung surface, and then combining these solutions to obtain a composite velocity field to help restore strong local discontinuities along the lung boundary.

### 2.2 Deep learning-based medical image registration

Existing deep learning-based medical image registration methods are mainly divided into supervised learning-based methods, unsupervised learning-based methods, and generative adversarial network (GAN)-based methods (19). Supervised learning-based registration methods require the use of real deformation fields or deformation fields simulated for training supervision signals. These include fully supervised learning methods that require deformation field labels, as well as weakly supervised learning methods that rely on other related information labels. These methods typically use architectures such as CNNs to directly learn the displacement vector field (DVF) from a pair of input images. They have achieved state-of-the-art performance in the registration of medical images from various organs, including the lungs (20), brain (21), abdomen (22), and prostate (23). Unsupervised learning-based registration methods do not rely on any form of ground truth data. Instead, they train the network by minimizing the difference between the fixed image and the transformed moving image, typically using image feature matching and similarity metrics as loss functions (24). One of the earliest unsupervised learning-based registration methods, VoxelMorph (25), parametrizes the mapping from input image pairs to deformation fields using CNNs. Subsequent works, inspired by this pioneering work, such as TransMorph (26), experimented with several hybrid architectures combining Transformers and CNNs. These studies confirmed the effectiveness of Transformer architectures in the field of medical image registration. Generative adversarial network (GAN)-based registration methods use a generator network to predict the deformation field while employing a discriminator network to evaluate the similarity between the deformed image and the fixed image. Adversarial training is used to enhance the quality of the deformation field (27, 28). Although supervised registration methods, which have accurate labels, achieve the best training results, their limitations are significant, whether they use deformation fields obtained from traditional methods or artificially simulated synthetic deformation fields for supervised training. To reduce the dependency on real deformation field labels, weakly supervised registration methods that use indirect reference labels have been widely adopted. For example, Hering et al. (9) employed multiple constraints to penalize unrealistic deformations, using a multi-scale framework to progressively refine the registration and calculate deformation fields at different scales to handle large deformations. Additionally, they applied volume change control to penalize image folding more strictly than regularization methods. It is worth noting that traditional methods are also applied in many deep learning approaches, especially in the regularization terms of loss functions designed based on different assumptions (29, 30). However, current research in medical image registration, particularly in lung CT image registration, mainly focuses on addressing deformation issues arising from multiple images, such as lung tissue movement caused by respiration and organ sliding. These studies aim to improve registration accuracy, particularly when dealing with deformations caused by respiratory motion, as well as 2D–3D reconstruction or inter-modal reconstruction.

### 2.3 Unsupervised learning

Unsupervised learning is a training strategy in machine learning, where the core idea is to directly mine the inherent features, potential relationships, or patterns from the data without the need for manually annotated labels. However, precise medical image registration typically requires domain experts to manually annotate corresponding points or structures for registration. This annotation process is often time-consuming, labor-intensive, and costly. Moreover, annotations may vary due to subjective differences in expert judgment, leading to annotation bias. Therefore, compared to supervised learning, unsupervised learning can take advantage of the distribution characteristics of large amounts of unlabeled data, saving the need for high-quality data annotation and reducing associated costs. It has been widely applied in current medical image registration research. One classic medical registration model, VoxelMorph (25), is an unsupervised registration model based on the UNet architecture. The input to the model is the reference image (also known as the fixed image, *I*_*f*_) and the moving image (also known as the moving image, *I*_*m*_). The model's output is the registered image (also known as the warped image, *I*_*w*_). The registration process calculates a deformation vector field (DVF) based on feature matching between *I*_*f*_ and *I*_*m*_ and then transforms *I*_*m*_ using the DVF to obtain *I*_*w*_. Currently, most unsupervised models follow the same workflow as VoxelMorph, where the features of the two images are first learned, spatial feature matching positions are sought, and similarity losses are used to optimize the model. The DVF is calculated to map from the space of *I*_*f*_ to that of *I*_*m*_, and the final warped image is obtained through the deformation calculation. The advantage of unsupervised learning in medical image registration lies in its ability to leverage the distribution characteristics of large amounts of unlabeled data, thus avoiding the high costs and subjective biases associated with manual annotation. For instance, by learning from vast amounts of unlabeled medical image data, the model can automatically discover anatomical structures and feature patterns in the images, enabling automated registration. Additionally, unsupervised learning methods can improve the model generalization ability through techniques like data augmentation, allowing it to better adapt to images from different patients and imaging conditions. Through this process, unsupervised learning in medical image registration enables efficient and automated registration, providing strong support for disease diagnosis, treatment monitoring, and prognostic evaluation.

## 3 Methods

### 3.1 The overall structure of the VoxelMorph model

VoxelMorph is a deep learning-based framework for medical image registration, the core idea of which is to utilize deep neural networks to directly predict the deformation vector field (DVF) between a pair of images. The model employs an encoder-decoder architecture, enabling efficient processing of image registration tasks. Comprising an encoder and a decoder, VoxelMorph leverages the DVF generated by the decoder to warp the moving image, thereby aligning it spatially with the reference image within a unified coordinate system.

The encoder component progressively extracts high-level features from the input image through a series of convolutional and pooling layers. Each convolutional block generally comprises two convolutional layers, each followed by a nonlinear activation function–commonly the Rectified Linear Unit. The convolution operation applies a set of learnable filters to the input, enabling the network to capture spatial hierarchies and local patterns, such as edges and textures. Subsequently, pooling operations are employed to downsample the spatial dimensions of the resulting feature maps, thereby reducing computational complexity, enhancing translational invariance, and facilitating the extraction of more abstract, higher-level features. This hierarchical processing allows the network to build increasingly complex representations of the input data. The specific formula is as follows:


(1) $$\begin{array}{l} E\left(x\right)=\text{MaxPool}\left(\text{ReLU}\left(\phi_{3\times 3}\left(\text{ReLU}\left(\phi_{3\times 3}\left(x\right)\right)\right)\right)\right) \end{array}$$

Here, ϕ_3 × 3_ represents the convolution operation using a 3 × 3 convolutional kernel, ReLU is the nonlinear activation function, and MaxPool refers to the max pooling operation. Each layer of the encoder progressively extracts local features of the image and captures higher-level feature abstractions in the deeper layers of the network.

The decoder part gradually restores the spatial resolution of the feature maps through upsampling and convolution operations. Each layer of the upsampling operation typically uses transposed convolution (also known as deconvolution) to increase the spatial dimensions of the feature maps. Each layer of the decoder receives features from the corresponding encoder layer via skip connections, preserving low-level feature information. The specific formula is as follows:


(2) $$\begin{array}{l} D\left(x\right)={\text{ConvTranspose}}_{3\times 3}\left(\text{ReLU}\left(\phi_{3\times 3}\left(\text{ReLU}\left(\phi_{3\times 3}\left(x\right)\right)\right)\right)\right) \end{array}$$

where, = ConvTranspose_3 × 3_ represents the upsampling operation using a 3 × 3 transposed convolution kernel. The output of the decoder is a deformation vector field (DVF), which describes how to map each voxel in the moving image to its corresponding position in the fixed image.

The ultimate goal of VoxelMorph is to deform the moving image into the space of the fixed image using the predicted deformation field. Specifically, for a given moving image *I*_*m*_ and fixed image *I*_*f*_, the moving image *I*_*m*_ is deformed into the warped image *I*_*w*_ through the deformation field (DVF), such that *I*_*w*_ aligns as closely as possible with *I*_*f*_. The application process for the deformation field is as follows:


(3) $$\begin{array}{l} I_{w}\left(i\right)=I_{m}\left(i+DVF\left(i\right)\right) \end{array}$$

where *i* represents the position in the image, and DVF(i) represents the deformation vector at position *i*. Linear interpolation is applied to handle non-integer coordinate values, ensuring that the deformed image *I*_*w*_ aligns with the fixed image *I*_*f*_.

VoxelMorph adopts an unsupervised learning strategy, training the model by minimizing the similarity loss between the fixed image and the deformed image. Through this unsupervised learning approach, VoxelMorph can learn the optimal deformation field based on the similarity between the images without the need for real deformation field labels, thus achieving efficient image registration.

### 3.2 Spatial and channel hybrid attention fusion module

To encourage the model to better achieve semantic fusion alignment and learn the potential relationships between the target image and the deformation field, we designed the Spatial and Channel Hybrid Attention Fusion (scHAF) Module. This module employs a hybrid strategy along the skip connection path to capture spatial feature attention and channel feature attention from shallow features. It retains the more registration-relevant information from the shallow features, enabling the semantic fusion alignment of deep and shallow features, and facilitating the optimization of the deformation field by the model.

**Figure 1:**
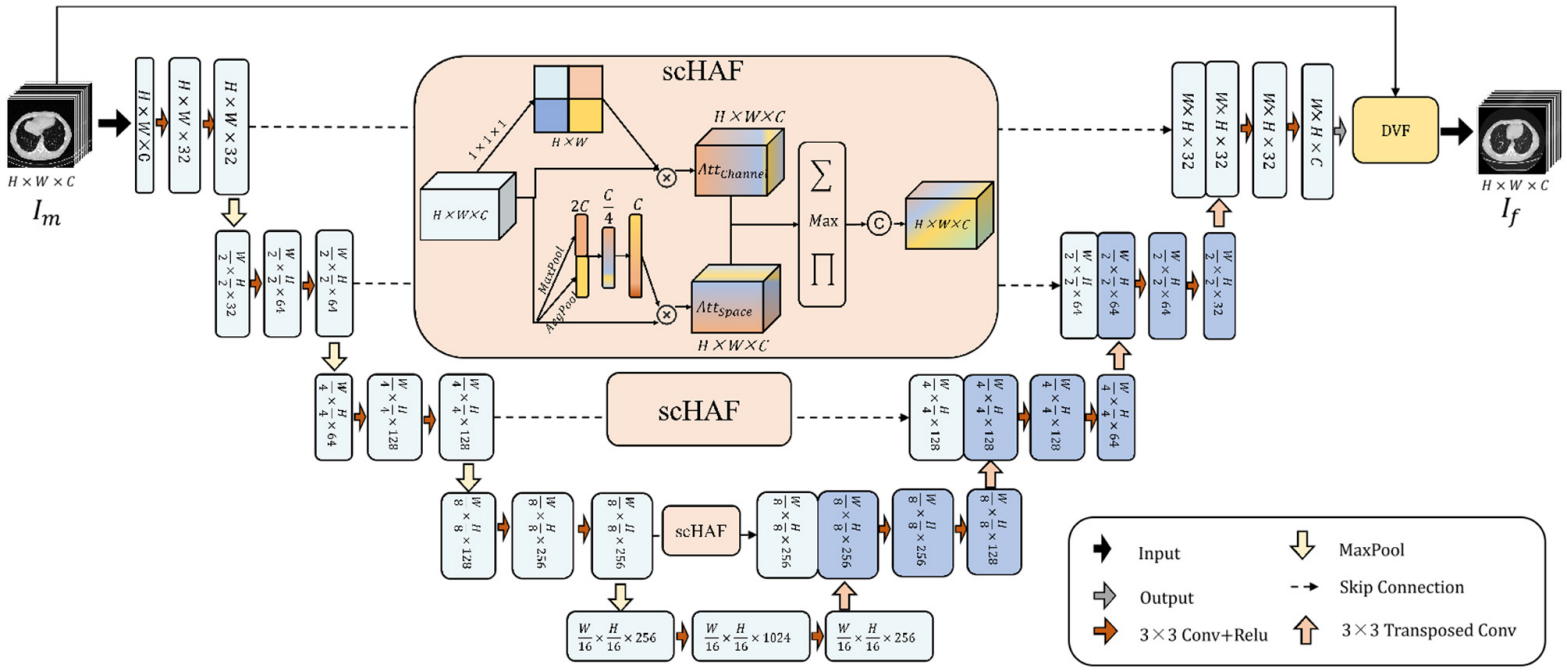
The architecture of UDRNet. The model takes a moving image, Im, as input. It performs feature extraction through successive convolutional and downsampling operations, and subsequently accomplishes feature recovery via successive upsampling and transposed convolutional operations. At the skip connection points, scHAF (spatially-adaptive feature filtering and fusion module) is employed for feature filtering and fusion. Ultimately, the model outputs a deformation field that represents the mapping from Im to the fixed image, If. The moving image Im is then warped using this deformation field to produce the registered image.

As Figure 1 shows, we first perform a 1 × 1 × 1 convolution on the shallow feature *F* ∈ ℝ^*H*×*W*×*C*^ to obtain a feature map of size *H*×*W*. Through a broadcasting operation, the channel feature attention $Att_{Channel}\in {\mathbb{R}}^{H\times W\times C}$ is applied, and then the shallow features *F* are multiplied pointwise with the channel-weighted attention to get the channel-weighted feature $F_{Channel}\in {\mathbb{R}}^{H\times W\times C}$.

At the same time, we perform global pooling and average pooling on the shallow features, resulting in vectors *MP* ∈ ℝ^1 × 1 × *C*^ and *AP* ∈ ℝ^1 × 1 × *C*^, respectively. These two vectors represent the most prominent and average features of the shallow features *F* along each channel dimension. We concatenate the vectors *MP* and *AP* to obtain the vector *MPAP* ∈ ℝ^1 × 1 × 2*C*^. Then, we apply two layers of MLP to compress and restore the features of *MPAP*. Finally, through a broadcasting operation, we obtain the spatial attention $Att_{Space}\in {\mathbb{R}}^{H\times W\times C}$, which is multiplied pointwise with *F* to get the spatially weighted feature $F_{Space}\in {\mathbb{R}}^{H\times W\times C}$.

$F_{Channel}\in {\mathbb{R}}^{H\times W\times C}$ and $F_{Space}\in {\mathbb{R}}^{H\times W\times C}$ strengthen the importance of shallow features in different spatial locations and channels, respectively. To fuse them, we designed a hybrid fusion strategy. As shown in Equation 4, for each position in *F*_*Channel*_ and *F*_*Space*_, we apply three operations: pointwise multiplication, pointwise addition, and pointwise maximum. The results of these three operations are concatenated along the channel dimension. Finally, a 3 × 3 convolution operation is applied to reshape the dimensions, obtaining the mixed feature.


(4) $$\begin{array}{r} F_{Hybrid}=Conv_{3\times 3}(Concate([\prod \left(F_{Channel},F_{Space}\right),\\ \sum \left(F_{Channel},F_{Space}\right),Max(F_{Channel},F_{Space})])) \end{array}$$

## 4 Experiment and results

### 4.1 Dataset

We conducted performance validation of our model on the CT Lung Registration dataset (31). The CT Lung Registration dataset was part of a task in the Learn2Reg 2022 challenge, and it contains 30 3D CT lung images collected from the Department of Radiology at the Radboud University Medical Center. Out of these, 20 CT images were assigned to the training set, and 10 CT images were assigned to the test set. The training set also includes the lung region segmentation mask for each CT image and automatically detected anatomical keypoints for guiding deformation field learning. The test set contains manually labeled keypoints by clinical experts, which serve as the benchmark for evaluating registration accuracy. The main purpose of this dataset is to study large deformations of the lungs during the breathing process. As the lungs undergo significant non-rigid deformation during the breathing cycle, some lung areas may not be fully visible in certain expiration phase CT scans (e.g., due to scanning range or patient position), making precise registration necessary.

We performed preprocessing operations such as windowing, normalization, and image cropping on the dataset to minimize noise interference and reduce the impact of differences in scanning devices or parameters. Below is a detailed introduction to the preprocessing operations.

1. Windowing: By adjusting the Window Width (WW) and Window Level (WL) of the lung CT images, the grayscale range of the lungs and related tissues is emphasized, allowing the model to more clearly observe target structures such as lung parenchyma, blood vessels, and nodules. The windowing operation is shown in Equation 1, where *I*_HU_ represents the CT value of each pixel in the original CT image. After the windowing operation, the pixel values corresponding to all pixels in the CT image are mapped to the range [0, 255].

$$I_{\text{windowed}}=\{\begin{array}{ll} 0, & \text{if }I_{\text{HU}}\leq WL-\frac{WW}{2}\\ 255, & \text{if }I_{\text{HU}}\geq WL+\frac{WW}{2}\\ 255\times \frac{I_{\text{HU}}-(WL-\frac{WW}{2})}{WW}, & \text{otherwise} \end{array}$$

2. Normalization: to enhance the contrast of the lungs and related tissues, highlighting the differences between different tissues, and to accelerate the model learning process, we performed a min-max normalization operation on all CT images, mapping the pixel values of each CT image to the range [0, 1]. The process of min-max normalization is shown in Equation 2, where *I*_norm_ represents the normalized pixel value of each pixel, *I*_windowed_ represents the pixel value after windowing, and *max*() and *min*() represent the functions for calculating the maximum and minimum values, respectively.

$$I_{norm}=\frac{I_{\text{windowed}}-min\left(I_{\text{windowed}}\right)}{max\left(I_{\text{windowed}}\right)-min\left(I_{\text{windowed}}\right)}$$

3. Image Cropping: To reduce the impact of regions outside the region of interest for registration and to lower the computational burden, thus accelerating the optimization process, we performed the final step of data preprocessing by cropping the original data from 192 × 192 × 208 to 192 × 192 × 192. The cropped areas correspond to the initial and final CT images in the patient's CT sequence, which are not relevant to the registration task.

### 4.2 Implementation details

We implement our network by Python 3.10.16, Pytorch 2.2.2 and train our network 150 epochs on NVIDIA GeForce RTX 4090 24G. Adam optimizer with a learning rate set to 1*e*−5 is selected to optimize our network. And if the test loss does not descend within five epochs, we will terminate the training in advance to avoid overfitting. Meanwhile, the Batch Size is set to 16, and the random number seed is 666.

### 4.3 Evaluation metrics

We choose to use three metrics: Dice Similarity Coefficient (Dice), Mean Squared Error (MSE), and Normalized Cross-Correlation (NCC) to evaluate the model performance in image registration. Below is a detailed introduction to the calculation and meaning of these three evaluation metrics.


(5) $$\begin{array}{l} \text{Dice}\left(A,B\right)=\frac{2|A\cap B|}{\left|A|+|B\right|} \end{array}$$

The calculation of the Dice coefficient is shown in Equation 5, where *A* and *B* represent the masks of the regions to be registered before and after registration, respectively. This metric measures the overlap between the segmented regions of the two images, with a value range of [0, 1]. The larger the value, the better the registration performance.


(6) $$\begin{array}{l} \text{MSE}\left(I_{f},I_{m}\right)=\frac{1}{N}\overset{N}{\underset{i=1}{\sum }}{\left(I_{f}\left(i\right)-I_{m}\left(i\right)\right)}^{2} \end{array}$$

The calculation of the *MSE* (Mean Squared Error) is shown in Equation 6, where *I*_*f*_ is the fixed image, *I*_*m*_ is the moving image, *N* is the total number of pixels, and *I*_*F*_(*i*) and *I*_*B*_(*i*) represent the grayscale values of the corresponding pixel in the two images. This metric calculates the mean squared difference in pixel values between the two images, with a smaller value indicating better registration accuracy.


(7) $$\begin{array}{l} \text{NCC}\left(I_{f},I_{m}\right)=\frac{\underset{i}{\sum }\left(I_{f}\left(i\right)-\mu_{f}\right)\left(I_{m}\left(i\right)-\mu_{m}\right)}{\sqrt{\underset{i}{\sum }{\left(I_{f}\left(i\right)-\mu_{f}\right)}^{2}\underset{i}{\sum }{\left(I_{m}\left(i\right)-\mu_{m}\right)}^{2}}} \end{array}$$

The calculation of the *NCC* (Normalized Cross-Correlation) is shown in Equation 7, where *I*_*f*_ is the fixed image, *I*_*m*_ is the moving image, *N* is the total number of pixels, *I*_*f*_(*i*) and *I*_*m*_(*i*) represent the grayscale values of the corresponding pixel in the two images, and μ_*f*_ and μ_*m*_ are the mean grayscale values of the fixed and moving images, respectively. This metric is used to assess the linear correlation of the grayscale patterns between the two images, with a range of [−1, 1]. A higher value indicates better image alignment, and *NCC* = 1 means the two images are perfectly aligned.

### 4.4 Comparison with other methods

In the comparative experiments, we conducted a comprehensive performance evaluation of the proposed unsupervised learning-based deformable lung CT image registration model and compared it with several advanced registration methods. Table 1 shows the average performance comparison on all validation set data, while Table 2 presents the average performance comparison on the top five best-performing data. The best results are marked in bold. The experimental results indicate that our model outperforms or is comparable to other advanced registration methods, such as 3D AttUNet (32), 3D ResUNet (33), 3D VoxelMorph (25), and 3D TransUNet (34). Specifically, our model achieves a Dice score of 54.92%, a 1-MSE score of 89.90%, and an NCC score of 91.49%, demonstrating significant advantages in registration accuracy and robustness.

**Table 1:** Comparison of our models with other registration models.

**Method**	**1-MSE (%)**	**NCC (%)**	**Dice (%)**
3D AttUNet (32)	88.91	89.72	54.13
3D ResUNet (33)	89.60	91.08	54.55
3D VoxelMorph (25)	89.72	91.36	54.67
3D TransUNet (34)	89.89	91.45	54.90
Ours	**89.90**	**91.49**	**54.92**

**Table 2:** Comparison of our models with other registration models (TOP 5).

**Method**	**1-MSE (%) (TOP 5)**	**NCC (%) (TOP 5)**	**Dice (%) (TOP 5)**
3D AttUNet (32)	90.86	90.54	58.34
3D ResUNet (33)	91.56	91.84	58.91
3D VoxelMorph (25)	91.65	92.04	58.99
3D TransUNet (34)	91.79	92.13	59.20
Ours	**91.82**	**92.27**	**59.27**

**Figure 2:**
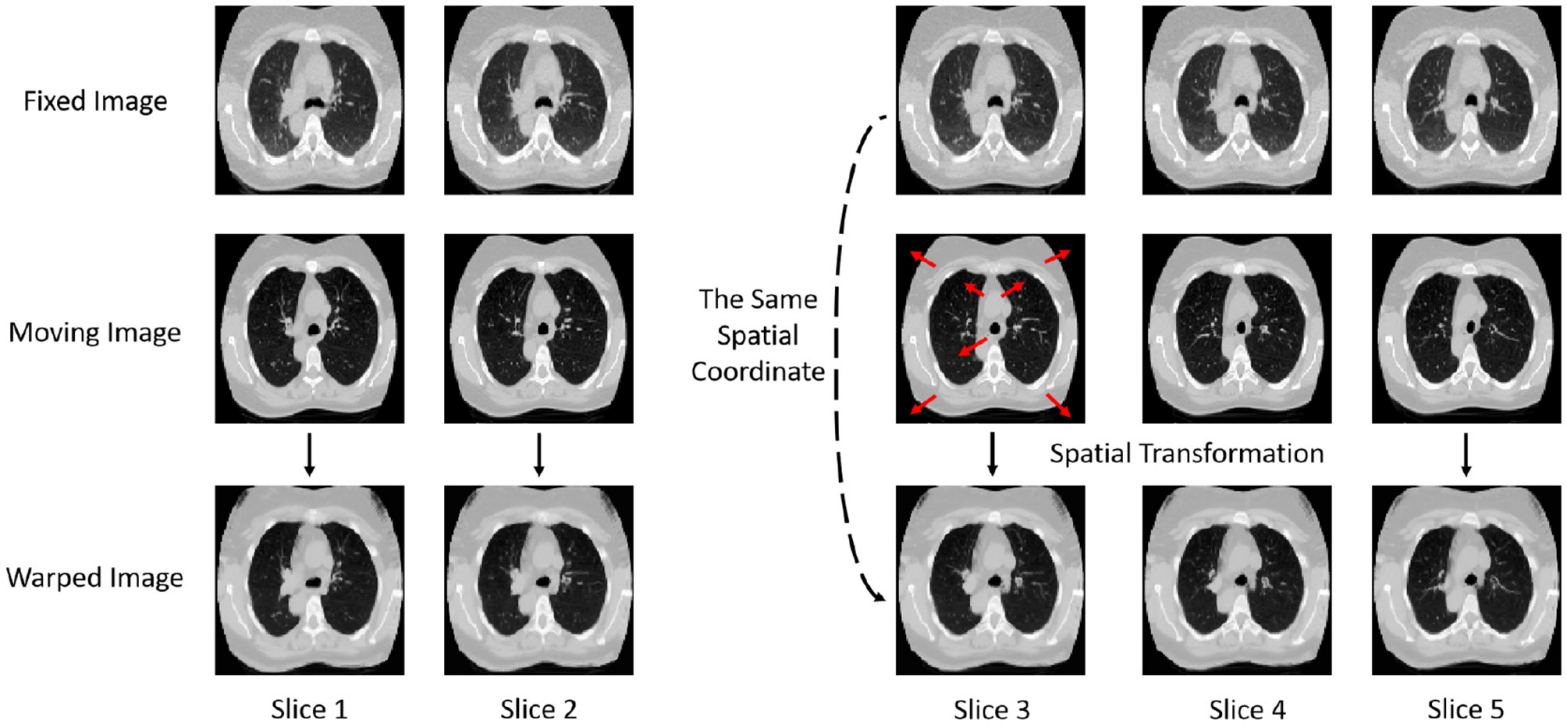
Display of images before and after registration. Comparison of fixed, moving, and warped images across multiple CT slices.

The images before and after registration are shown in Figure 2. The displayed registration results clearly present the corresponding slices of the fixed image, moving image, and warped image for the same patient. These images visually illustrate the process in which the moving image is adjusted to align with the fixed image in the same spatial coordinate system through spatial transformation. From the figure, it can be observed that the model effectively adjusts the deformation in the moving image through precise spatial transformations, resulting in a high degree of spatial alignment between the two images. This alignment is not only well-presented in terms of the macroscopic structure but also shows high consistency in fine details, indicating that the registration model exhibits excellent accuracy and robustness. Such precise registration is of significant importance for subsequent medical image analysis and clinical applications, providing reliable imaging support for disease diagnosis, treatment monitoring, and prognosis assessment.

### 4.5 Ablation study

In the ablation study, we compared the performance of the VoxelMorph model before and after adding the scHAF module, with the experimental results shown in Table 3. The results demonstrate that the model performance improved across all metrics after incorporating the scHAF module. Specifically, 1-MSE increased from 89.72 to 89.90%, NCC improved from 91.36 to 91.49%, and Dice rose from 54.67 to 54.92%. These results indicate that the introduction of the scHAF module significantly enhanced the registration accuracy and robustness of the VoxelMorph model.

**Table 3:** Comparison of our models with scHAF and without scHAF.

**Method**	**1-MSE (%)**	**NCC (%)**	**Dice (%)**
3D VoxelMorph (25)	89.72	91.36	54.67
Ours (3D VoxelMorph + scHAF)	**89.90**	**91.49**	**54.92**

The scHAF module, by mixing and fusing shallow spatial and channel features at the skip connections, better achieves semantic fusion alignment and learns the potential correlation between the image to be registered and the deformation field. Specifically, the scHAF module first extracts channel feature attention and spatial feature attention from shallow features and then fuses these two features through a hybrid strategy. This fusion strategy not only retains the information most relevant to the registration from the shallow features but also enhances the model ability to capture features through the weighted consideration of both channel and spatial dimensions. Experimental results show that the introduction of the scHAF module significantly improves the model registration performance, especially when dealing with complex lung CT images. It enables the model to more accurately capture structural changes in the lungs, thus enhancing both the accuracy and robustness of the registration process.

## 5 Conclusion

In current medical image registration research, despite significant progress made by deep learning methods in certain tasks, there are still several challenges. First, traditional unsupervised registration methods often overlook the dynamic changes in pathological areas within images, leading to registration results that fail to accurately reflect real biological changes. Secondly, existing registration methods face difficulties in achieving high-precision registration when handling lung CT images due to the complexity and elasticity of lung tissue, as well as issues like inconsistent imaging device parameters and pose deviations. These challenges limit the effectiveness and reliability of registration techniques in clinical applications.

To address these issues, we propose an unsupervised learning-based deformable lung CT image registration model and design the Spatial and Channel Hybrid Attention Fusion (scHAF) module. The scHAF module mixes and fuses shallow spatial and channel features at the skip connection, enabling better semantic alignment and learning the potential correlations between the image to be registered and the deformation field. Specifically, the scHAF module first extracts channel feature attention and spatial feature attention from the shallow features, then fuses these two features using a hybrid strategy. This approach retains more relevant information in the shallow features and enhances the model ability to capture key characteristics. Our baseline model adopts the VoxelMorph, which is based on an encoder-decoder structure. The encoder gradually extracts high-level features from the image using multiple layers of convolution and pooling operations, while the decoder restores the spatial resolution of the feature maps using upsampling and convolution operations. By designing and incorporating the scHAF module, the model can more effectively handle complex deformations in lung CT images, improving both registration accuracy and robustness.

The experimental results show that our model outperforms or is comparable to several advanced registration methods across multiple metrics. These results indicate that the introduction of the scHAF module improves the registration performance of the model, particularly when handling complex lung CT images, where it can more accurately capture the changes in lung structure, thereby enhancing both the accuracy and robustness of the registration process. Therefore, our model and the scHAF module provide an effective solution for unsupervised lung CT image registration, with broad application potential.

## Data Availability

Publicly available datasets were analyzed in this study. This data can be found at: https://learn2reg.grand-challenge.org/.
